# Characterizing the Manifest Probability Distributions of Three Latent Trait Models for Accuracy and Response Time

**DOI:** 10.1007/s11336-019-09668-3

**Published:** 2019-03-27

**Authors:** M. Marsman, H. Sigurdardóttir, M. Bolsinova, G. Maris

**Affiliations:** 1 University of Amsterdam, Nieuwe Achtergracht 129B, PO Box 15906, 1001 NK Amsterdam, The Netherlands; 2 Tilburg University, Tilburg, The Netherlands; 3 ACTNext, Iowa City, USA

**Keywords:** drift diffusion model, Dutch identity, graphical model, hierarchical model, item response theory, response times, signed residual time model, conditional independence

## Abstract

In this paper we study the statistical relations between three latent trait models for accuracies and response times: the hierarchical model (HM) of van der Linden (Psychometrika 72(3):287–308, 2007), the signed residual time model (SM) proposed by Maris and van der Maas (Psychometrika 77(4):615–633, 2012), and the drift diffusion model (DM) as proposed by Tuerlinckx and De Boeck (Psychometrika 70(4):629–650, 2005). One important distinction between these models is that the HM and the DM either assume or imply that accuracies and response times are independent given the latent trait variables, while the SM does not. In this paper we investigate the impact of this conditional independence property—or a lack thereof—on the manifest probability distribution for accuracies and response times. We will find that the manifest distributions of the latent trait models share several important features, such as the dependency between accuracy and response time, but we also find important differences, such as in what function of response time is being modeled. Our method for characterizing the manifest probability distributions is related to the Dutch identity (Holland in Psychometrika 55(6):5–18, 1990).

## Introduction

In this paper we wish to study the statistical relations between three latent trait models for accuracies and response times: the hierarchical model (HM) of van der Linden (2007), the signed residual time model (SM) of Maris and van der Maas (2012), and the drift diffusion model (DM) proposed by Tuerlinckx and De Boeck (2005). These models come from different backgrounds and differ in many respects. A key distinction between the three latent trait models is in the relation they stipulate between accuracy and response time after conditioning on the latent variables. Whereas responses are independent of response times after conditioning on the latent variables in both the DM and the HM, this is not the case for the SM. The conditional independence property has received much attention in the psychometric literature on response time modeling (e.g., Bolsinova, De Boeck, & Tijmstra, 2010; Bolsinova & Maris, 2016; Bolsinova & Tijmstra, 2016; Bolsinova, Tijmstra, & Molenaar, 2017; van der Linden & Glas, 2017).

The way that these and other latent trait models for accuracy and response time are related has been the topic of several publications (e.g., Molenaar, Tuerlinckx, & van der Maas, 2017a; 2015b; van Rijn & Ali, 2015a), but what sets our approach apart from earlier comparison attempts is that we do not work with their latent trait formulations. A serious complication with comparing latent trait models is that they are usually not defined on a common metric or space. As a result, it is unclear what the conditional independence property, for example, says about the distribution of observables, or how one can compare latent variables and their impact on observables across models. The manifest distribution —i.e., the distribution of observables after having integrated out the latent variables—does not suffer from these complications and is easily compared. We therefore work with manifest distributions in this paper.

The comparison of manifest probability distributions crucially depends on having their analytic expressions available to us, but unfortunately, this is not the case for the latent trait models that we study here. To overcome this complication, we reverse-engineer an approach that was originally used by Kac (1968) to find a latent variable expression of a graphical model known now as the Ising model (Ising, 1925). The work of Kac has revealed a broad equivalence between psychometric item response models and network models from statistical physics (Epskamp, Maris, Waldorp, & Borsboom, 2018; Marsman et al., 2018; Marsman, Tanis, Bechger, & Waldorp, 2019). Here we use it to characterize the manifest distribution of latent trait models that are in the exponential family. Another way to express the manifest distributions of latent trait models is the Dutch identity (Holland, 1990). Our approach and the Dutch identity are, of course, very much related, and we will study this relation in detail.

The remainder of this paper is structured as follows: In the next section, we formally introduce the three latent trait models. We will focus on versions of the latent trait models that either use or imply the two-parameter logistic model for the marginal distribution of response accuracies—i.e., the conditional distribution of accuracies given the latent variables after having integrated out the response times. After having introduced the three latent trait models we introduce our approach for characterizing their manifest probability distributions. Here we will also study the relation between our approach and the Dutch identity. We then characterize and analyze the manifest probability distributions that are implied by the three latent trait models. Our paper ends with a discussion of these results.

## Models

Before we introduce the three latent trait models we first wish to introduce some notation and clarify our terminology. We will assume that the item parameters of the latent trait models are fixed constants, but that the accuracies, the response times, and the latent variables are random. Since these variables are assumed to be random everywhere in this paper, we do not distinguish between (vectors of) random variables and their realizations. We will use $\mathbf {x}$ to denote a vector of *p* response accuracies—$x_i \in \{0,\,1\}$—and use $\mathbf {t}$ to denote a vector of *p* response times—$t_i \in \mathbb {R}^+$ for the HM and DM and $t_i \in (0,\,\text {d}_i)$ for the SM, see below. The two latent variables ability and speed will be denoted with $\theta$ and $\eta$, respectively. Finally, we will use “marginal distribution” to refer to the conditional distribution of one type of observable, e.g.,

$$\begin{aligned} p(x \mid \theta ,\,\eta ) = \int _{\mathbb {R}^+} p(x,\,t\mid \theta ,\,\eta ) \,\text {d}t, \end{aligned}$$

where the other observables have been integrated out, and we will use “manifest distribution” to refer to distributions of the form

$$\begin{aligned} p(x,\,t) = \int _{\mathbb {R}}\int _{\mathbb {R}} p(x,\,t \mid \theta ,\,\eta ) \, p(\theta ,\,\eta )\,\text {d}\theta \,\text {d}\eta , \end{aligned}$$

where the latent variables have been integrated out.

### The Hierarchical Model

The HM, as proposed by van der Linden (2007), is a general statistical framework for modeling accuracies and responses times that is based on the idea that there are two latent traits at work; ability $\theta$ governs the response accuracy distribution and speed $\eta$ the response time distribution. Importantly, the response accuracy distribution is assumed to be independent of speed $\eta$ given ability $\theta$, the response time distribution is assumed to be independent of ability $\theta$ given speed $\eta$, and it is also assumed that accuracies and response times are independent given the full set of latent traits, i.e.,

$$\begin{aligned} p(\mathbf {x} ,\,\mathbf {t} \mid \theta ,\,\eta ) = p(\mathbf {x} \mid \theta ) \, p(\mathbf {t} \mid \eta ). \end{aligned}$$

This setup provides a plug-and-play framework for modeling accuracy and response times: The measurement model for ability $\theta$ —marginal distribution of accuracies $p(\mathbf {x} \mid \theta )$—can be chosen independently of the measurement model for speed $\eta$—marginal distribution of response times $p(\mathbf {t} \mid \eta )$. The HM is concluded with a model for the two latent traits.

Different measurement models for ability $\theta$ have been used in the literature. For example, van der Linden (2007) used normal ogive models, Bolsinova, De Boeck, and Tijmstra (2017) used their logistic counterparts, while Zhan, Jiao, and Liao (2018) used cognitive diagnosis models instead. Here, we use the two-parameter logistic model,

1 $$\begin{aligned} p(\mathbf {x} \mid \theta ) = \prod _{i=1}^p p(x_i \mid \theta ) = \prod _{i=1}^p \frac{\exp \left( x_i\, \alpha _i(\theta + \beta _i)\right) }{1+\exp \left( \alpha _i(\theta + \beta _i)\right) }, \end{aligned}$$

where $\alpha _i$ is an item discrimination parameter and $\beta _i$ is an item easiness parameter. There are two reasons for using the two-parameter logistic model here. Firstly, the marginal distribution of accuracies $p(\mathbf {x} \mid \theta )$ that is implied by the versions of the SM and DM that are used here is also a two-parameter logistic model. Secondly, the two-parameter logistic model is a member of the exponential family of distributions, which will be convenient for expressing the manifest probability distribution $p(\mathbf {x} ,\,\mathbf {t})$.

Different measurement models for speed $\eta$ have also been used in the literature. For example, van der Linden (2006) used a log-normal distribution, Fox, Klein Entink, and van der Linden (2007) used a linear factor model for log-transformed response times, and Klein Entink, van der Linden, and Fox (2009) used models based on Box–Cox transformations of response times. In this paper we will use the log-normal distribution,

2 $$\begin{aligned} p(\mathbf {t} \mid \eta ) = \prod _{i=1}^p p(t_i \mid \eta ) = \prod _{i=1}^p \sqrt{\frac{\phi _i}{2\pi }}\,\frac{1}{t_i}\, \exp \left( -\frac{1}{2}\, \phi _i\, \left( \ln (t_i) + \eta - \xi _i\right) ^2\right) , \end{aligned}$$

where $\xi _i$ is an item time intensity parameter and $\phi _i$ an item precision parameter. The item precision $\phi _i$ is the response time analogue of the item discrimination in the two-parameter logistic model; larger values of $\phi _i$ imply that speed explains a larger portion of the log-time variance. The log-normal distribution is a common choice for the measurement model for speed $\eta$ in the HM framework and is also a member of the exponential family of distributions.

To conclude the HM we specify a distribution for ability $\theta$ and speed $\eta$. Typically, a bivariate normal distribution is used in which ability and speed are correlated. To identify the model, however, the means of the bivariate normal need to be constrained to zero and the marginal variance of ability needs to be constrained to one.^1^

### The Signed Residual Time Model

The SM has been proposed by Maris and van der Maas (2012) as a measurement model for ability $\theta$ in the context of tests with item-level time limits. The model was specifically designed for tests that use the following scoring rule,

$$\begin{aligned} \text {s} = \sum _{i=1}^p\text {s}_i=(2x_i- 1 )(\text {d}_i-t_i), \end{aligned}$$

where $\text {d}_i$ is the time limit for item *i*. This scoring rule encourages persons to work fast but punishes guessing: Residual time $\text {d}_i - t_i$ is gained when the response is correct, but is lost when the response is incorrect. Van Rijn and Ali (2017b) demonstrated that the SM is also appropriate for applications where these time limits are not specified a priori, but “estimated” from the observed response time distributions.

The SM specifies the following distribution for accuracy $\mathbf {x}$ and response times $\mathbf {t}$:

3 $$\begin{aligned} p(\mathbf {x},\,\mathbf {t}\mid \theta ) = \prod _{i=1}^p (\theta +\beta _i) \frac{\exp \left( (2x_i-1)(\text {d}_i-t_i)(\theta + \beta _i)\right) }{\exp (\text {d}_i(\theta +\beta _i))-\exp (-\text {d}_i(\theta +\beta _i))}, \end{aligned}$$

where $\beta _i$ is an item easiness parameter. Observe that the SM is an exponential family model and that the scoring rule $\text {s}$ is the sufficient statistic for ability $\theta$. The SM has been generalized by van Rijn and Ali (2017b) allowing the items to differ in their discriminative power even when the time limits are the same across items. In this paper we will use the standard version of the SM.

Whereas the HM characterizes the joint distribution of accuracies and response times by specific choices of the marginals $p(\mathbf {x} \mid \theta )$ and $p(\mathbf {t} \mid \eta )$, the SM directly specifies a joint distribution for accuracies and response times $p(\mathbf {x},\,\mathbf {t} \mid \theta )$. By integrating out the response times we obtain the marginal distribution for accuracies $p(\mathbf {x} \mid \theta )$. Maris and van der Maas (2012) show that this marginal distribution is the two-parameter logistic model in Eq. (1), where the item discrimination $\alpha _i$ is equal to the item time limit $\text {d}_i$. In a similar way, we obtain the marginal distribution of response times $p(\mathbf {t} \mid \theta )$ by summing out the accuracies,

$$\begin{aligned} p(\mathbf {t} \mid \theta ) = \prod _{i=1}^p (\theta + \beta _i)\frac{\exp ((\text {d}_i - t_i)(\theta + \beta _i))+\exp (-(\text {d}_i-t_i)(\theta +\beta _i))}{\exp (\text {d}_i(\theta +\beta _i))-\exp (-\text {d}_i(\theta +\beta _i))}. \end{aligned}$$

An alternative specification of the SM is in terms of accuracies and what Maris and van der Maas (2012, p. 624) refer to as *pseudo*-response times $\mathbf {t}^*$. *Pseudo*-response times are obtained from response times through the transformation

4 $$\begin{aligned} t^*_i = {\left\{ \begin{array}{ll} t_i &{} \text { if }x_i = 1,\\ \text {d}_i - t_i &{} \text { if }x_i = 0. \end{array}\right. } \end{aligned}$$

This transformation from response times to *pseudo*-response times is one-to-one, so that no information is lost. One convenient feature of using *pseudo*-response times instead of response times is that the *pseudo*-response times and accuracies are (conditionally) independent in the SM, i.e.,

$$\begin{aligned} p(\mathbf {x},\,\mathbf {t}^*\mid \theta ) = p(\mathbf {x}\mid \theta )p(\mathbf {t}^*\mid \theta ), \end{aligned}$$

where the marginal distribution $p(\mathbf {t}^*\mid \theta )$ is equal to,

5 $$\begin{aligned} p(\mathbf {t}^*\mid \theta ) = \prod _{i=1}^p (\theta +\beta _i)\,\frac{\exp \left( -t_i^*\left( \theta +\beta _i\right) \right) }{1- \exp \left( -\text {d}_i\left( \theta +\beta _i\right) \right) }. \end{aligned}$$

### The Drift Diffusion Model

The DM was introduced by Ratcliff (1978) as a model for two-choice experiments. In the DM, evidence for either choice accumulates over time until a decision boundary is reached. One way to characterize this evidence accumulation process is in terms of a Wiener process with constant drift and volatility, and absorbing upper and lower boundaries (Cox & Miller, 1970). The drift $\mu$ of the diffusion process determines how fast information is accumulated, the volatility $\sigma$ determines how noisy the accumulation process is, and the distance between the two boundaries $\alpha$ determines how much evidence needs to be accumulated before a choice is made. The process has two additional parameters: a bias parameter *z* that indicates the distance from the starting point to the lower boundary, and the non-decision time $T_{(er)}$. A commonly used simplification of the DM assumes that the process is unbiased $z = \frac{1}{2}\alpha$.

The DM has been extended to model differences between persons and tasks. For example, Tuerlinckx and De Boeck (2005) proposed to decompose the drift $\mu$ of the accumulation process into a person and an item part —i.e., $\mu = \theta + \beta _i$—and to treat the distance between the boundaries as an item characteristic —i.e., $\alpha =\alpha _i$. The person component $\theta$ in the drift specification carries the interpretation of an ability in item response theory models, as a higher value of $\theta$ implies an increased probability of choosing the correct alternative. To identify the DM, Tuerlinckx and De Boeck (2005) fixed the volatility $\sigma$ to one. The joint distribution of decision times and the chosen alternatives —i.e., response accuracies if the upper and the lower boundaries correspond to the correct and incorrect responses—is then equal to:

6 $$\begin{aligned} p(\mathbf {x},\,\mathbf {t}\mid \theta )&= \prod _{i = 1}^p \frac{\pi }{\alpha _i^2}\exp \left( \frac{1}{2}\alpha _i\left( 2x_i-1\right) (\theta + \beta _i) - \frac{1}{2}\left( t_i-T_{(er)}\right) (\theta + \beta _i)^2\right) \nonumber \\&\quad \times \sum _{n = 1}^{\infty } \sin \left( \frac{1}{2}\pi n\right) \exp \left( -\frac{1}{2\alpha _i^2}\pi ^2n^2\left( t_i-T_{(er)}\right) \right) . \end{aligned}$$

Both the SM and the DM directly specify a joint distribution of accuracies and decision times (response times) that is based on one latent trait, ability $\theta$. In contrast to the SM, however, accuracies and response times are independent given ability in the (unbiased) DM. The marginal $p(\mathbf {x} \mid \theta )$ is the two-parameter logistic model in Eq. (1), where the discrimination parameter equals the distance between the boundaries in the diffusion process, and the easiness parameter is an item effect on drift of the diffusion process. The marginal $p(\mathbf {t} \mid \theta )$ is equal to

$$\begin{aligned} p(\mathbf {t} \mid \theta )&= \prod _{i = 1}^p \frac{2\pi }{\alpha _i^2} \cosh \left( \frac{1}{2}\alpha _i(\theta + \beta _i)\right) \exp \left( -\frac{1}{2}\left( t_i-T_{i}^{(er)}\right) (\theta + \beta _i)^2\right) \\&\quad \times \sum _{n = 1}^{\infty } \sin \left( \frac{1}{2}\pi n\right) \exp \left( -\frac{1}{2\alpha _i^2}\pi ^2n^2\left( t_i-T_{i}^{(er)}\right) \right) . \end{aligned}$$

Even though the marginal distribution $p(\mathbf {x} \mid \theta )$ is a member of the exponential family, neither the marginal distribution $p(\mathbf {t}\mid \theta )$ nor the joint distribution $p(\mathbf {x},\,\mathbf {t}\mid \theta )$ is a member of the exponential family. The primary reason that the latter cannot be written in exponential family form is because it implies a statistic $\text {s}_1 = \text {s}_1(x,\,t) = \sum _i\alpha _i\,x_i -\sum _{i}\beta _i\,t_i$ that is sufficient for $\theta$ and another statistic $\text {s}_2 = \text {s}_2(t) = \sum _it_i$ that is sufficient for $-\frac{1}{2}\theta ^2$. If we express the latter as $\eta = \eta (\theta ) = -\frac{1}{2}\theta ^2$, we end up with an exponential family model subject to constraints on the parameters $\theta$ and $\eta$: $\eta$ is functionally related to ability $\theta$. This is known as a curved exponential family model (Efron, 1975, 1978).

## Characterizing Manifest Probabilities of Latent Trait Models

We consider the general case of an *item response theory (IRT)* model for accuracy and response times in an exponential family form:

7 $$\begin{aligned} p(\mathbf {x},\,\mathbf {t} \mid \varvec{\zeta }) = \prod _{i=1}^p p(x_i,\,t_i \mid \varvec{\zeta }) = \prod _{i=1}^p\frac{1}{\text {Z}_i(\varvec{\zeta })} \, \text {b}_i \,\exp \left( \,\mathbf {s}_i^\mathsf{T} \varvec{\zeta } \right) , \end{aligned}$$

where $\mathbf {s}_i=\mathbf {s}_i(x_i,\,t_i)$ is a (possibly vector-valued) statistic that is sufficient for the (possibly vector-valued) latent variable $\varvec{\zeta }$, and the function $\text {b}_i = \text {b}_i(x_i,\,t_i)$ is a base measure that does not depend on the value of this latent variable. The base measure serves as the probability measure when the exponential term in Eq. (7) is given no weight, i.e., when $\varvec{\zeta } = \mathbf {0}$. Finally, the function $\text {Z}_i(\varvec{\zeta })$ is a normalizing constant that is defined as

$$\begin{aligned} \text {Z}_i(\varvec{\zeta }) = \sum _{x_i=0}^1 \int _{\mathbb {R}^+} \text {b}_i(x_i,\,t_i) \,\exp \left( \,\mathbf {s}_i(x_i,\,t_i)^\mathsf{T} \varvec{\zeta } \right) \,\text {d}t_i, \end{aligned}$$

which, when it exists, ensures that the probabilities add up to one.

We will make use of the fact that the three latent trait models can be written in the form of Eq. (7), and outline an approach for expressing the manifest distribution for latent trait models of this form. But since Eq. (7) ignores any functional relation that may exist between its latent variables, a variant of our approach needs to be used to express the manifest distribution for the DM. We will point out how our approach can be used for models, such as the DM, that can be written in the form

8 $$\begin{aligned} p(\mathbf {x},\,\mathbf {t} \mid \theta ) = \prod _{i=1}^p p(x_i,\,t_i \mid \theta ) = \prod _{i=1}^p\frac{1}{\text {Z}_i(\theta )} \, \text {b}_i \,\exp \left( \,s_{i1}\theta - s_{i2}\frac{1}{2}\theta ^2\right) . \end{aligned}$$

For latent trait models that are of the form of Eq. (7) or Eq. (8) we can make use of the following latent variable distribution to express its manifest distribution.

### Definition 1

For models $p(\mathbf {x},\,\mathbf {t} \mid \varvec{\zeta })$ that are of the form of Eq. (7) or Eq. (8) we may define the latent variable distribution

9 $$\begin{aligned} g(\varvec{\zeta }) =\frac{1}{\text {Z}} \prod _{i=1}^p \text {Z}_i(\varvec{\zeta })\, k(\varvec{\zeta }), \end{aligned}$$

where $k(\varvec{\zeta })$ is a kernel density and $\text {Z}$ is the normalizing constant of $g(\varvec{\zeta })$. For every kernel distribution $k(\varvec{\zeta })$ for which the normalizing constant $\text {Z}$ is finite, i.e.,

$$\begin{aligned} 0< \text {Z} = \int _{\Omega _{\varvec{\zeta }}}\prod _{i=1}^p \text {Z}_i(\varvec{\zeta }) \, k(\varvec{\zeta }) \,\text {d}\varvec{\zeta } < \infty , \end{aligned}$$

where $\Omega _{\varvec{\zeta }}$ is the support of $\varvec{\zeta }$, $g(\varvec{\zeta })$ is a valid probability distribution.

The distribution in Definition 1 was inspired by the latent trait distribution that has been introduced with the latent variable expression of a graphical model from physics known as the Ising (1925) model by Kac (1968, see also Marsman et al., 2018; Epskamp, Maris, Waldorp, & Borsboom, 2018), but a similar construction can also be found in, for instance, Cressie and Holland (1983, Eq. A9) and McCullagh (1994). We can now state our first result.

### Theorem 1

When $p(\mathbf {x} ,\,\mathbf {t}\mid \varvec{\zeta })$ is of the form of Eq. (7) and the latent variable distribution $g(\varvec{\zeta })$ in Eq. (9) is a valid probability distribution, then the manifest distribution $p(\mathbf {x},\,\mathbf {t})$ is given by

$$\begin{aligned} p(\mathbf {x},\,\mathbf {t}) = \frac{1}{\text {Z}} \prod _{i=1}^p\text {b}_i\,\mathbb {E}\left( \prod _{i=1}^p\exp \left( \mathbf {s}_i^\mathsf{T}\varvec{\zeta }\right) \right) , \end{aligned}$$

where $\text {Z}$ is a normalizing constant and the expectation is an integral with respect to the kernel density $k(\varvec{\zeta })$.

We omit the simple proof of Theorem 1, which requires one to fill in the definitions of the models in Eqs. (7) and (9), and then integrate out the latent variable $\varvec{\zeta }$. In a similar way, the manifest distribution for latent trait models that are of the form of Eq. (8) can be expressed as

10 $$\begin{aligned} p(\mathbf {x},\,\mathbf {t}) = \frac{1}{\text {Z}} \prod _{i=1}^p\text {b}_i\,\mathbb {E}\left( \prod _{i=1}^p\exp \left( s_{i1}\theta - s_{i2}\frac{1}{2}\theta ^2\right) \right) . \end{aligned}$$

Theorem 1 shows that for any latent trait model of the form of Eq. (7), combined with a latent variable distribution of the form of Eq. (9), the manifest distribution can be characterized in terms of the base measures $\text {b}_i$ and the moment generating function of the kernel distribution $k(\varvec{\zeta })$. This is similar to Holland’s (1990) Dutch identity, which was initially formulated for locally independent binary response models by Holland (1990) and extended to a locally dependent response model by Ip (2002) and a polytomous response model by Hessen (2012). The following theorem gives an extension of the Dutch identity for response models that are of the exponential family form of Eq. (7). Its proof is in the Appendix.

### Theorem 2

(The Dutch identity) Suppose that $p(\mathbf {x},\,\mathbf {t})$ is of the form

$$\begin{aligned} p(\mathbf {x},\,\mathbf {t}) = \int _{\Omega _\zeta } p(\mathbf {x},\,\mathbf {t} \mid \varvec{\zeta })\, f(\varvec{\zeta }) \text { d}\varvec{\zeta }, \end{aligned}$$

where $\Omega _{\varvec{\zeta }}$ is the support of $\varvec{\zeta }$, and $p(\mathbf {x},\,\mathbf {t} \mid \varvec{\zeta })$ is of the form of Eq. (7). Then for any vector $\mathbf {y} \in \Omega _{\mathbf {x}}$ and $\mathbf {w} \in \Omega _{\mathbf {t}}$, where $\Omega _{\mathbf {x}}$ and $\Omega _{\mathbf {t}}$ denote the support of $\mathbf {x}$ and $\mathbf {t}$, respectively, we have

$$\begin{aligned} \frac{p(\mathbf {x},\,\mathbf {t})}{p(\mathbf {y},\,\mathbf {w})} = \prod _{i=1}^p \frac{\text {b}_i(x_i,\,t_i)}{\text {b}_i(y_i,\,w_i)} \mathbb {E}\left( \left. \prod _{i=1}^p \exp \left( \left[ \mathbf {s}_i(x_i,\,t_i) - \mathbf {s}_i(y_i,\,w_i)\right] ^\mathsf{T} \varvec{\zeta }\right) \,\right| \, \mathbf {S} = \sum _i\mathbf {s}_i(y_i,\,w_i)\right) , \end{aligned}$$

where the expectation is an integral with respect to the posterior density $f(\theta \mid \mathbf {S} = \sum _i\mathbf {s}_i(y_i,\,w_i))$.

It is easy to verify that whenever $f(\varvec{\zeta })$ is of the form of $g(\varvec{\zeta })$ in Eq. (9), the identity in Theorem 2 reduces to

$$\begin{aligned} \frac{p(\mathbf {x},\,\mathbf {t})}{p(\mathbf {y},\,\mathbf {w})} = \prod _{i=1}^p \frac{\text {b}_i(x_i,\,t_i) }{\text {b}_i(y_i,\,w_i)} \times \frac{\mathbb {E}\left( \prod _{i=1}^p\exp \left( \mathbf {s}_i(x_i,\,t_i)^\mathsf{T} \varvec{\zeta }\right) \right) }{\mathbb {E}\left( \prod _{i=1}^p\exp \left( \mathbf {s}_i(y_i, w_i)^\mathsf{T} \varvec{\zeta }\right) \right) }, \end{aligned}$$

which was to be expected from Theorem 1. Observe that in this case the expectations imply integrating with respect to the kernel density $k(\varvec{\zeta })$ that is used to define $g(\varvec{\zeta })$ in Eq. (9).

Both Theorems 1 and 2 characterize the manifest distribution in terms of a moment generating function, and for their practical application it is important to find a convenient form for this moment generating function. The Dutch identity, for example, has provided a general analytic solution for the (extended) Rasch model (Cressie & Holland, 1983; Tjur, 1982), but to come to an analytic expression for other latent trait models an assumption has to be made about the posterior distribution of the latent variable. In a similar way, we have to choose a kernel $k(\varvec{\zeta })$ for the practical application of Theorem 1. The following corollary shows how a multivariate normal kernel distribution $k(\varvec{\zeta })$ can be used to express the manifest distribution in a simple analytic form.

### Corollary 1

If $p(\mathbf {x},\,\mathbf {t}\mid \varvec{\zeta })$ is of the form in Eq. (7), and the latent variable distribution $g(\varvec{\zeta })$ is of the form in Eq. (9) with a multivariate normal kernel $k(\varvec{\zeta })$ having a mean vector $\mathbf {m}$ and covariance matrix $\mathbf {V}$, then (i)

11 $$\begin{aligned} p(\mathbf {x},\,\mathbf {t}) = \frac{1}{\text {Z}} \exp \left( \sum _{i=1}^p \ln \text {b}_i + \left[ \sum _{i=1}^p \mathbf {s}_i\right] ^\mathsf{T}\mathbf {m} + \frac{1}{2}\left[ \sum _{i=1}^p \mathbf {s}_i\right] ^\mathsf{T}\mathbf {V}\left[ \sum _{i=1}^p \mathbf {s}_i\right] \right) , \end{aligned}$$

where $\text {Z}$ is a normalizing constant, and (ii) the posterior distribution $g(\varvec{\zeta } \mid \mathbf {s}_1,\,\dots ,\,\mathbf {s}_p)$ is multivariate normal with mean vector $\mathbf {m} + \mathbf {V}\left[ \sum _{i=1}^p\mathbf {s}_i\right]$ and covariance matrix $\mathbf {V}$.

We will omit the proof of Corollary 1, which requires one to insert in Theorem 1 the moment generating function of the multivariate normal distribution, i.e.,

$$\begin{aligned} \mathbb {E}\left( \exp \left( \mathbf {r}^\mathsf{T}\varvec{\zeta }\right) \right) = \exp \left( \mathbf {r}^\mathsf{T}\mathbf {m} + \frac{1}{2}\mathbf {r}^\mathsf{T}\mathbf {V}\mathbf {r}\right) , \end{aligned}$$

using $\mathbf {r} = \sum _i\mathbf {s}_i$ as the interpolating parameter vector. Since we cannot make use of this moment generating function for the curved exponential family models in Eq. (8), the normal kernel does not result in the same simple form for these models. We can, however, make use of the following identity

$$\begin{aligned} \mathbb {E}\left( \exp \left( r_{1}\theta - r_{2}\frac{1}{2}\theta ^2\right) \right) = \sqrt{\frac{v^2}{r_{2}\,v^2+1}}\, \exp \left( \frac{r_{1}^2\,v^2 + 2r_{1}\,m - r_{2}m^2}{2r_{2}\,v^2 + 2}\right) , \end{aligned}$$

where the expectation is taken with respect to a normal distribution with mean *m* and variance $v^2$. Inserting this identity into the expression of the manifest distribution in Eq. (10) with $r_1=\sum _is_{1i} = s_{1+}$ and $r_2=\sum _is_{2i}=s_{2+}$, we end up with the following expression

12 $$\begin{aligned} p(\mathbf {x},\,\mathbf {t}) = \frac{1}{\text {Z}} \prod _{i=1}^p\text {b}_i\,\sqrt{\frac{v^2}{s_{2+}\,v^2+1}}\, \exp \left( \frac{s_{1+}^2\,v^2 + 2s_{1+}\,m - s_{2+}m^2}{2s_{2+}\,v^2 + 2}\right) . \end{aligned}$$

This is a relatively simple analytic expression when we set *m* to zero. We will use Corollary 1 and Eq. (12) to characterize the manifest probability distributions of the three latent trait models.

Corollary 1 mirrors the results for assuming posterior normality of the latent trait in combination with the Dutch identity as evidenced in Corollary 1 of Holland (1990), Corollary 1 of Ip (2002), and Theorem 2 of Hessen (2012), see also the log-multiplicative association models of Anderson and Vermunt (2000), and Anderson and Yu (2007), and the fused latent and graphical IRT model of Chen, Li, Liu, and Ying (2018).

There appears to be a deeper connection between the prior assumption that leads to our Corollary 1 and the posterior assumption that leads to Corollary 1 of Holland (1990). What we know is that the latent variable distribution in Eq. (9) with a normal kernel is one way to ensure posterior normality of the latent variables for any latent trait model of the form of Eq. (7). To see why this is the case, consider the posterior with a prior distribution $f(\varvec{\zeta }) = g(\varvec{\zeta })$ of the form of Eq. (9),

$$\begin{aligned} g(\varvec{\zeta } \mid \mathbf {s})&\propto \prod _{i=1}^p\frac{1}{\text {Z}_i(\varvec{\zeta })} \,e^{\left[ \mathbf {s}_i(y_i,\,w_i)^\mathsf{T} \varvec{\zeta }\right] } g(\varvec{\zeta }) \\&\propto \prod _{i=1}^p\frac{1}{\text {Z}_i(\varvec{\zeta })} \,e^{\left[ \mathbf {s}_i(y_i,\,w_i)^\mathsf{T} \varvec{\zeta }\right] } \prod _{i=1}^p\text {Z}_i(\varvec{\zeta })\, k(\varvec{\zeta })\\&= \exp \left( \left[ \sum _{i=1}^p\mathbf {s}_i(y_i,\,w_i)^\mathsf{T}\right] \varvec{\zeta }\right) \, k(\varvec{\zeta }), \end{aligned}$$

which is proportional to a multivariate normal distribution if and only if the kernel $k(\varvec{\zeta })$ is a multivariate normal distribution. The reverse need not be true since there exists at least one counterexample: Suppose that $\mathbf {s}_i$ follows a multivariate normal distribution with a mean equal to $\varvec{\zeta }$ (or some linear function of $\varvec{\zeta }$), such that $p(\mathbf {s}_i \mid \varvec{\zeta })$ can be written in the form of Eq. (7), then a normal prior distribution for $\varvec{\zeta }$, which is not of the form of Eq. (9), also ensures posterior normality of the latent variables. It may well be the case that this is the only exception to the general correspondence between the prior assumption that leads to our Corollary 1 and the posterior assumption that leads to Corollary 1 of Holland (1990).

## The Manifest Probabilities of the Three Latent Trait Models

In this section we use Corollary 1 and Eq. (12) to characterize the manifest distributions for the three latent trait models. For the HM and SM we will use Corollary 1 to generate a manifest distribution over the realizations of some vector random variable $\mathbf {q}$ that is of the form

13 $$\begin{aligned} p(\mathbf {q}) = \frac{1}{\text {Z}} \exp \left( \mathbf {q}^\mathsf{T}\varvec{\mu } + \mathbf {q}^\mathsf{T}\varvec{\Sigma }\,\mathbf {q}\right) \,\omega (\mathbf {q}), \end{aligned}$$

where $\varvec{\mu }$ denotes a vector of intercepts, $\varvec{\Sigma }$ denotes a symmetric matrix of pairwise interactions, $\omega (\mathbf {q})$ denotes a base measure that serves as the probability measure when the pairwise interactions are equal to zero, and $\text {Z}$ denotes the model’s normalizing constant. For the DM we will use Eq. (12) to generate a manifest distribution over the realizations of a random vector $\mathbf {q}$ that is of the form

14 $$\begin{aligned} p(\mathbf {q}) = \frac{1}{\text {Z}} \exp \left( \mathbf {q}^\mathsf{T}\varvec{\mu } + h(\mathbf {q})\,\mathbf {q}^\mathsf{T}\varvec{\Sigma }\,\mathbf {q}\right) \,\omega (\mathbf {q}), \end{aligned}$$

which resembles the manifest distribution in Eq. (13), except that the pairwise interactions are now “weighted” by *h*. What we shall see is that the three latent trait models fundamentally differ in what the random variable $\mathbf {q}$ is, revealing key differences in the function of response time they are modeling, but also that accuracies and responses times are dependent in the manifest distribution of each latent trait model, except for fringe cases that are uncommon in practice. We will now consider each of the models in turn.

### The Hierarchical Model

The version of the HM that is considered here is of the form

$$\begin{aligned} p\left( \mathbf {x},\,\mathbf {t} \mid \varvec{\zeta } = (\theta ,\, \eta )^\mathsf{T}\right) \,g\left( \varvec{\zeta } = (\theta ,\, \eta )^\mathsf{T}\right) = p(\mathbf {x}\mid \theta )\, p(\mathbf {t} \mid \eta )\, g(\theta ,\,\eta ), \end{aligned}$$

where the marginal $p(\mathbf {x} \mid \theta )$ is the two-parameter logistic model introduced in Eq. (1), the marginal $p(\mathbf {t} \mid \eta )$ is the log-normal distribution introduced in Eq. (2), and $g(\theta ,\,\eta )$ is of the form of Eq. (9) using a bivariate normal kernel distribution $k(\theta ,\,\eta )$ for ability $\theta$ and speed $\eta$, using a mean vector $\mathbf {m} = \mathbf {0}$ and covariance matrix

$$\begin{aligned} \mathbf {V} = \begin{pmatrix} 1 &{} \rho \, v_\eta \\ \rho \, v_\eta &{} v_\eta ^2 \end{pmatrix}, \end{aligned}$$

where $\rho$ denotes the *a priori* correlation between ability and speed, and $v_\eta ^2$ the *a priori* variance of speed.

To come to an expression for the manifest probability distribution of this version of the HM we first rewrite the conditional distribution of accuracies and response times $p(\mathbf {x},\,\mathbf {t} \mid \theta ,\,\eta )$ to fit the form of Eq. (7). To this aim, we introduce the statistic

$$\begin{aligned} \mathbf {s}_i = \mathbf {s}_i(x_i,\,t_i) = \begin{pmatrix} x_i\alpha _i \\ -\ln (t_i)\,\frac{1}{2}\phi _i \end{pmatrix}, \end{aligned}$$

the base measures

$$\begin{aligned} \text {b}_i = \text {b}_i(x_i,\,t_i) = \exp \left( x_i\alpha _i\beta _i - \ln (t_i)^2\,\frac{1}{2}\phi _i - \ln (t_i) \, (1-\phi _i\xi _i)\right) , \end{aligned}$$

and the normalizing constants

$$\begin{aligned} \text {Z}_i(\theta ,\,\eta )&= \frac{\sqrt{2\pi }}{\sqrt{\phi _i}}\,\exp \left( \frac{1}{2}\,\phi _i\,(\eta - \xi _i)^2\right) \,\left\{ 1 + \exp \left( \alpha _i(\theta +\beta _i)\right) \right\} . \end{aligned}$$

Having expressed the conditional distribution of accuracies and response times in the form of Eq. (7), we can now apply Corollary 1 to obtain the manifest distribution. It is convenient to characterize this manifest distribution in terms of log-transformed response times $u_i = \ln (t_i)$ instead of response times. The manifest probability distribution of accuracies and log-transformed response times that results is equal to

$$\begin{aligned} p(\mathbf {x},\,\mathbf {u})&= \frac{1}{\text {Z}} \exp \left( \begin{pmatrix}\mathbf {x} \\ \mathbf {u}\end{pmatrix}^\mathsf{T} \begin{pmatrix}\varvec{\alpha }\odot \varvec{\beta } \\ \varvec{\phi } \odot \varvec{\xi } \end{pmatrix} + \frac{1}{2} \begin{pmatrix} \mathbf {x}\\ \mathbf {u} \end{pmatrix}^\mathsf{T} \begin{pmatrix} \varvec{\alpha } \, \varvec{\alpha }^\mathsf{T} &{} - \rho \, v_\eta \, \varvec{\alpha }\, \varvec{\phi }^\mathsf{T}\\ - \rho \, v_\eta \, \varvec{\phi }\, \varvec{\alpha }^\mathsf{T} &{} v_\eta ^2\,\varvec{\phi }\,\varvec{\phi }^\mathsf{T} \end{pmatrix} \begin{pmatrix} \mathbf {x}\\ \mathbf {u} \end{pmatrix}\right) \\&\quad \prod _{i=1}^p \exp \left( -\frac{1}{2}\phi _i \,u_i^2\right) , \end{aligned}$$

where $\odot$ refers to Hadamard product. Observe that this manifest distribution is of the form of Eq. (13) for a random variable $\mathbf {q} = (\mathbf {x}^\mathsf{T}, \mathbf {u}^\mathsf{T})^\mathsf{T}$, with intercepts

$$\begin{aligned} \varvec{\mu } = \begin{pmatrix}\varvec{\alpha }\odot \varvec{\beta }\\ \varvec{\phi } \odot \varvec{\xi } \end{pmatrix}, \end{aligned}$$

a rank two matrix of pairwise interactions

$$\begin{aligned} \varvec{\Sigma } = \frac{1}{2} \begin{pmatrix} \varvec{\alpha } \, \varvec{\alpha }^\mathsf{T} &{} - \rho \, v_\eta \, \varvec{\alpha }\, \varvec{\phi }^\mathsf{T}\\ - \rho \, v_\eta \, \varvec{\phi }\, \varvec{\alpha }^\mathsf{T} &{} v_\eta ^2\,\varvec{\phi }\,\varvec{\phi }^\mathsf{T} \end{pmatrix} = \frac{1}{2} \begin{pmatrix} \varvec{\alpha } \\ v_\eta \, \varvec{\phi } \end{pmatrix} \begin{pmatrix} 1 &{} -\rho \\ -\rho &{} 1 \end{pmatrix} \begin{pmatrix} \varvec{\alpha } \\ v_\eta \, \varvec{\phi } \end{pmatrix}^\mathsf{T}, \end{aligned}$$

and a normal base measure

$$\begin{aligned} \omega (\mathbf {u}) = \prod _{i=1}^p \omega _i(u_i) = \prod _{i=1}^p \exp \left( -\frac{1}{2}\phi _i \,u_i^2\right) , \end{aligned}$$

with precisions $\phi _i$. In this model the associations, or pairwise interactions, between accuracies and log-response times, are of the opposite sign of the correlation $\rho$ between the two latent variables of the HM: Faster responses correspond to correct answers when $\rho > 0$; slower responses correspond to correct answers when $\rho < 0$.

There are two important characteristics that can be observed from the manifest probability distribution of our version of the HM. Firstly, the base measure $\omega (\mathbf {u})$ that is used here stipulates an *a priori* restriction on the variance of the speed parameter $v_\eta ^2$ and the item-specific precisions $\phi _i$. To see this, observe that for this base measure the manifest distribution is a proper probability distribution—i.e., integrates to one—if and only if

$$\begin{aligned} v_\eta ^2 < \min _i\left( \frac{1}{\phi _i}\right) . \end{aligned}$$

Since the variance of the $\log (t_i)$ is equal to

$$\begin{aligned} \text {Var}(\log (t_i)) = \mathbb {E}(\text {Var}(\log (t_i) \mid \eta )) +\text {Var}(\mathbb {E}(\log (t_i) \mid \eta )) = \frac{1}{\phi _i} + v_\eta ^2, \end{aligned}$$

this restriction on $v_\eta ^2$ and $\phi _i$ implies that the speed variable can account for less than 50% of the total variance of the $\log (t_i)$.

A second important characteristic that can be observed from the manifest probability distribution of our version of the HM concerns the associations between accuracies and log-response times, which are encoded in the matrix of pairwise interactions $\varvec{\Sigma }$. First, note that when the interaction between an accuracy $x_i$ and log-transformed response time $u_i$ in the manifest distribution is equal to zero, these variables are independent conditional upon the remaining accuracies and log-transformed response times:Thus, $x_i$ and $u_i$ are conditionally independent whenever one of the following conditions apply: The correlation $\rho$ between speed and ability is zero; the discrimination $\alpha _i$ of item *i* is zero; the precision $\phi _i$ of item *i* is zero, and/or the *a priori* variance $v_\eta ^2$ of the speed variable is zero. The only non-trivial condition that leads to conditional independence between accuracy and response time is the *a priori* independence of ability and speed in the HM. But this entails the extreme case in which all of the accuracies are independent of all of the response times, which is unlikely to occur in psychometric practice.

### The Signed Residual Time Model

There are two versions of the SM that are considered here. The first version of the SM stipulates a distribution of accuracies and residual response times, and the second version of the SM stipulates a distribution of accuracies and *pseudo*-response times. We will first characterize the manifest probability distribution of accuracies and residual times and revert to *pseudo*-response times after that.

#### The Manifest Distribution of Accuracy and Residual Response Time

The SM was introduced in Eq. (3) and can be expressed in the exponential family form of Eq. (7) with statistics

$$\begin{aligned} \text {s}_i = \text {s}_i(x_i,\,t_i) = (2x_i-1)(\text {d}_i - t_i), \end{aligned}$$

base measures

$$\begin{aligned} \text {b}_i = \text {b}_i(x_i,\,t_i) = \exp \left( (2x_i-1)(\text {d}_i-t_i)\beta _i\right) , \end{aligned}$$

and normalizing constants

$$\begin{aligned} \text {Z}_i(\theta ) = \frac{\exp \left( \text {d}_i(\theta + \beta _i)\right) - \exp \left( -\text {d}_i(\theta +\beta _i)\right) }{\theta + \beta _i}. \end{aligned}$$

Having expressed the conditional distribution of accuracies and residual times of the SM in the form of Eq. (7) we can now use Corollary 1 to obtain their manifest distribution. Assuming a normal kernel with mean $m = 0$ and variance $v^2 = 1$, Corollary 1 leads us to the following manifest distribution,

$$\begin{aligned} p(\mathbf {x},\, \mathbf {t})= & {} \frac{1}{\text {Z}}\exp \left( \left[ (2\mathbf {x}-\mathbf {1}_p) \odot (\mathbf {d} - \mathbf {t})\right] ^\mathsf{T} \varvec{\beta } + \frac{1}{2}\left( \left( 2\mathbf {x} - \mathbf {1}_p\right) \odot \left( \mathbf {d} - \mathbf {t}\right) \right) ^\mathsf{T}\left( \left( 2\mathbf {x} - \mathbf {1}_p\right) \right. \right. \\&\quad \left. \left. \odot \left( \mathbf {d} - \mathbf {t}\right) \big )\right. \phantom {\left( \left[ (2\mathbf {x}-\mathbf {1}_p) \odot (\mathbf {d} - \mathbf {t})\right] ^\mathsf{T} \varvec{\beta } + \frac{1}{2}\left( \left( 2\mathbf {x} - \mathbf {1}_p\right) \odot \left( \mathbf {d} - \mathbf {t}\right) \right) ^\mathsf{T}\left( \left( 2\mathbf {x} - \mathbf {1}_p\right) \right. \right. }\right) , \end{aligned}$$

where $\mathbf {1}_p$ is the unit vector of length *p*. One way to write this distribution more succinctly is to express it in terms of the random variables $y_i = (2x_i-1)$ and residual times $r_i = \text {d}_i-t_i$, which gives

15 $$\begin{aligned} p(\mathbf {y},\,\mathbf {r}) = \frac{1}{\text {Z}}\exp \left( \left( \mathbf {y}\odot \mathbf {r}\right) ^\mathsf{T} \varvec{\beta } + \frac{1}{2}\left( \mathbf {y}\odot \mathbf {r}\right) ^\mathsf{T}\left( \mathbf {y}\odot \mathbf {r}\right) \right) . \end{aligned}$$

This is of the form of the manifest distribution in Eq. (13) for the random variable $\mathbf {q} = \mathbf {y}\odot \mathbf {r}$, with intercepts $\varvec{\mu } = \varvec{\beta }$, a rank one matrix of pairwise interactions $\varvec{\Sigma } = \frac{1}{2}\mathbf {1}_p\mathbf {1}_p^\mathsf{T}$, and a uniform base measure $\omega (\mathbf {q}) = 1$. In this model, larger residual times (faster responses) are associated with an increased probability that a person responds accurately to easy items ($\beta _i > 0$) and a decreased probability to respond accurately to difficult items ($\beta _i < 0$). The association between the response accuracies of different items is positive and increases with increasing residual response times (faster responses).

There are two important characteristics that can be observed from the manifest probability distribution of the SM. One characteristic appears when we view the manifest distribution as a distribution for the random variables $\mathbf {y}$, as this closely resembles a graphical model from physics that is known as the Ising model (Lenz, 1920; Ising, 1925). The Ising model is characterized by the following probability distribution over realizations of $\mathbf {y}$

$$\begin{aligned} p(\mathbf {y}) = \frac{1}{\text {Z}}\exp \left( \mathbf {y}^\mathsf{T}\varvec{\mu } + \mathbf {y} ^\mathsf{T}\varvec{\Sigma } \,\mathbf {y}\right) , \end{aligned}$$

where $\varvec{\mu }$ denotes a vector of *p* intercepts $\mu _i$, and $\varvec{\Sigma }$ is a symmetric $p \times p$ matrix of pairwise interactions $\sigma _{ij}$, similar to our Eq. (13). However, where the intercepts and interactions are fixed effects in the Ising network model, they are random effects here, with $\varvec{\mu } = \mathbf {r} \odot \varvec{\beta }$ and $\varvec{\Sigma } = \frac{1}{2}\mathbf {r}\mathbf {r}^\mathsf{T}$. This view of the SM thus provides a novel way for modeling the intercepts and matrix of associations in the Ising model.

A second important characteristic that can be observed from the manifest probability distribution of the SM concerns the association between accuracies and residual response times. Whereas we have found that for the manifest distribution of the HM that accuracy can be conditionally independent of response time in a non-trivial manner, at least in theory, this is not the case with the SM. This can be observed, for example, from the conditional distribution of accuracy and residual response time for an item *i* given the accuracies and residual times of the remaining items

$$\begin{aligned} p\left( y_i,\,r_i \mid \mathbf {y}^{(i)},\,\mathbf {t}^{(i)}\right) = \frac{\exp \left( y_ir_i\left[ \beta _i + \sum _{j \ne i} y_jr_j\right] \right) }{\frac{\exp \left( \beta _i + \sum _{j \ne i} y_jr_j\right) - \exp \left( -\beta _i - \sum _{j \ne i} y_jr_j\right) }{\beta _i + \sum _{j \ne i} y_jr_j}}, \end{aligned}$$

from which it is clear that there are no values of $\beta _i$ (or $\mathbf {y}^{(i)}$ and $\mathbf {r}^{(i)}$) that render $y_i$ and $r_i$ (conditionally) independent.

#### The Manifest Distribution of Accuracy and *Pseudo*-response Time

An alternative formulation of the SM is in terms of accuracies and *pseudo*-response times, which is of the form

$$\begin{aligned} p(\mathbf {x},\,\mathbf {t}^*\mid \theta ) = p(\mathbf {x}\mid \theta )\,p(\mathbf {t}^*\mid \theta ), \end{aligned}$$

where the marginal $p(\mathbf {x} \mid \theta )$ is the two-parameter logistic model in Eq. (1), and the marginal $p(\mathbf {t}^*\mid \theta )$ is given in Eq. (5).

To characterize the manifest distribution of accuracies and *pseudo*-response times for this version of the SM we can take two approaches. Firstly, we may express the conditional distribution of accuracies and *pseudo*-response times $p(\mathbf {x},\,\mathbf {t}^*\mid \theta )$ in the exponential family form of Eq. (7) and then apply Corollary 1 using a normal kernel distribution with mean $m =0$ and variance $v^2 = 1$ to this conditional distribution. Alternatively, we may rewrite the sufficient statistic for residual response times in the manifest distribution in Eq. (15) through the relation

$$\begin{aligned} \text {s}_i^*= x_i\text {d}_i - t_i^*= {\left\{ \begin{array}{ll} \text {d}_i - t_i &{} \text { if }x_i = 1\\ - (\text {d}_i-t_i) &{} \text { if }x_i = 0 \end{array}\right. } \Longleftrightarrow (2x_i-1)(\text {d}_i - t_i) = y_ir_i = \text {s}_i. \end{aligned}$$

Both approaches lead to the following manifest distribution of accuracies and *pseudo*-response times

$$\begin{aligned} {p(\mathbf {x},\,\mathbf {t}^*)} = {\frac{1}{\text {Z}} \exp }\left( \begin{pmatrix} {\mathbf {x}}\\ {\mathbf {t}^*} \end{pmatrix}^{\mathsf{T}}\begin{pmatrix}{\mathbf {d}\odot \varvec{\beta }}\\ {-\mathbf {1}_p \odot \varvec{\beta }} \end{pmatrix} {+ \frac{1}{2}}\begin{pmatrix} {\mathbf {x}}\\ {\mathbf {t}^*} \end{pmatrix}^{\mathsf{T}} \begin{pmatrix} \mathbf {d}\\ -\mathbf {1}_p \end{pmatrix} \begin{pmatrix} \mathbf {d}\\ -\mathbf {1}_p \end{pmatrix}^\mathsf{T} \begin{pmatrix} {\mathbf {x}}\\ {\mathbf {t}^*} \end{pmatrix} \right) , \end{aligned}$$

which is of the form of the manifest distribution in Eq. (13) for the random variable $\mathbf {q} = (\mathbf {x}^\mathsf{T},\,\mathbf {t}^{*\mathsf T})^\mathsf{T}$, with intercepts

$$\begin{aligned} \varvec{\mu } = \begin{pmatrix} \mathbf {d}\odot \varvec{\beta }\\ -\mathbf {1}_p\odot \varvec{\beta } \end{pmatrix}, \end{aligned}$$

a rank one matrix of pairwise interactions

$$\begin{aligned} \varvec{\Sigma } = \frac{1}{2}\begin{pmatrix} \mathbf {d}\\ -\mathbf {1}_p \end{pmatrix} \begin{pmatrix} \mathbf {d}\\ -\mathbf {1}_p \end{pmatrix}^\mathsf{T}, \end{aligned}$$

and a uniform base measure $\omega (\mathbf {q}) \propto 1$. Observe that for this model both the associations between accuracies and the associations between *pseudo*-response times are positive, yet the associations between accuracies and *pseudo*-response times are negative: Correct responses are associated with smaller *pseudo*-time values (faster response times); incorrect responses are associated with larger *pseudo*-time values (slower response times).

The manifest distribution of accuracies and *pseudo*-response times of the SM is of the same form as the manifest distribution of accuracies and the log-transformed response times of the HM, and thus they share certain characteristics. For example, both models share the following Markov property: When the association between an accuracy and a response time for an item *i* is equal to zero, then this implies that these two variables are independent conditional upon the remaining accuracies and response times,However, it is immediately clear that this association is never zero in practice, since $d_i = 0$ would imply a zero second time limit for item *i*.

### The Drift Diffusion Model

The version of the DM that is considered here—which was introduced in Eq. (6)—can be expressed in the form of Eq. (8), with statistics

$$\begin{aligned} \mathbf {s}_i(x_i,\,t_i) = \begin{pmatrix} \alpha _i\,x_i - \beta _i\,t_i\\ t_i \end{pmatrix}, \end{aligned}$$

base measures

$$\begin{aligned} \text {b}_i = \text {b}_i(x_i,\,t_i) = \exp \left( \alpha _i\,\beta _i\,x_i - \frac{1}{2}\,\beta _i^2\,t_i\right) \sum _{n = 1}^{\infty } \sin \left( \frac{1}{2}\pi n\right) \exp \left( -\frac{1}{2\alpha _i^2}\pi ^2n^2\left( t_i-T_{(er)}\right) \right) , \end{aligned}$$

and normalizing constants

$$\begin{aligned} \text {Z}_i(\theta ) = \frac{\alpha _i^2 }{ \pi } \exp \left( \frac{1}{2}\alpha _i(\theta +\beta _i)-\frac{1}{2}T_{(er)}(\theta + \beta _i)^2\right) . \end{aligned}$$

Having expressed the DM in the form of Eq. (8), we may now use Eq. (12) to express its manifest distribution. Assuming a latent trait distribution $g(\theta )$ of the form of Eq. (9) with a normal kernel with a mean $m = 0$ and variance $v^2 = 1$, Eq. (12) leads us to the following manifest distribution

$$\begin{aligned} {p(\mathbf {x},\,\mathbf {t}) = \frac{1}{\text {Z}} \exp }\left( \begin{pmatrix}{\mathbf {x}}\\ {\mathbf {t}}\end{pmatrix}^{\mathsf{T}} \begin{pmatrix} {\varvec{\alpha } \odot \varvec{\beta }}\\ {-\frac{1}{2}\varvec{\beta } \odot \varvec{\beta }} \end{pmatrix} {+ \frac{1}{2t_+ + 2} }\begin{pmatrix}{\mathbf {x}}\\ {\mathbf {t}}\end{pmatrix}^{\mathsf{T}} \begin{pmatrix}{\varvec{\alpha }}\\ {-\varvec{\beta }} \end{pmatrix} \begin{pmatrix}{\varvec{\alpha }}\\ {-\varvec{\beta }}\end{pmatrix}^{\mathsf{T}} \begin{pmatrix}{\mathbf {x}}\\ {\mathbf {t}}\end{pmatrix}\right) \, {\omega (\mathbf {t})} \end{aligned}$$

where $t_+ = \sum _it_i$, and $\omega (\mathbf {t})$ is a base measure,

$$\begin{aligned} \omega (\mathbf {t}) = \frac{1}{\sqrt{t_+ + 1}} \prod _{i=1}^p\sum _{n=1}^\infty \sin \left( \frac{1}{2}\pi n\right) \exp \left( -\frac{1}{2\alpha _i^2}\pi ^2n^2\left( t_i-T_{(er)}\right) \right) . \end{aligned}$$

This is of the form of the manifest distribution in Eq. (14) for the random variable $\mathbf {q} = \left( \mathbf {x}^\mathsf{T},\,\mathbf {t}^\mathsf{T}\right) ^\mathsf{T}$, that is characterized by the intercepts

$$\begin{aligned} \varvec{\mu } = \begin{pmatrix}{\varvec{\alpha } \odot \varvec{\beta }}\\ {-\frac{1}{2}\varvec{\beta } \odot \varvec{\beta }} \end{pmatrix}, \end{aligned}$$

a rank one matrix of pairwise interactions

$$\begin{aligned} \varvec{\Sigma } = \frac{1}{2} \begin{pmatrix}{\varvec{\alpha }}\\ {-\varvec{\beta }} \end{pmatrix} \begin{pmatrix}{\varvec{\alpha }}\\ {-\varvec{\beta }}\end{pmatrix}^{\mathsf{T}}, \end{aligned}$$

the weight function $h(\mathbf {t}) = (t_+ + 1)^{-1}$, and the aforementioned base measure.

There are two important characteristics that can be observed from the manifest probability distribution of our version of the DM. The first observation is that the association between both accuracies and response times is scaled by the total time $t_+$ that is spent on the test. This implies smaller associations between accuracies and response times for pupils that take longer to complete the test, and larger associations for pupils that take less time to complete the test. In none of the three other manifest probability distributions that were considered here have we seen an influence of the total time that was spent on the test.

A second observation is that since the interaction between accuracy and response time is scaled with the total test time, i.e.,

$$\begin{aligned} - x_i \, t_i \, \frac{1}{1+t_+}\, \frac{1}{2}\, \alpha _i \, \beta _i, \end{aligned}$$

and the same holds for the interactions between accuracies, i.e.,

$$\begin{aligned} x_i \,x_j \,\frac{1}{1+t_+}\,\frac{1}{2}\,\alpha _i\,\alpha _j, \end{aligned}$$

the accuracy of an item is related to all of the response times. There is only one way for the accuracy of an item to be conditionally independent from its response time in the manifest distribution, which is when the associated discrimination is equal to zero. However, the accuracy is then not only independent of all of the response times, but also of all remaining accuracies (and of ability in the latent trait formulation). As a consequence, accuracy and response time are conditionally independent only in a fringe case that we do not expect to see in psychometric practice.

## Discussion

The goal of this paper was the statistical comparison of three latent trait models for accuracy and response time: the hierarchical model (HM) of van der Linden (2007), the signed residual time model (SM) of Maris and van der Maas (2012), and the drift diffusion model (DM) as proposed by Tuerlinckx and De Boeck (2005). Our idea was to work with the manifest distributions of observables that were generated by these latent trait models, as they are more easily compared than their original latent trait formulations. To characterize these manifest distributions we have reverse-engineered an approach by Kac (1968), which inspired a new method for expressing manifest distributions. This method is summarized in our Theorem 1 and Corollary 1 and is related to the Dutch identity (Holland, 1990), which is our Theorem 2 for the response models considered in this paper. Our assumption of a normal kernel density for the latent trait parameters appeared to be closely related to the posterior normality assumption that is often used with the Dutch identity, but more importantly, it has allowed us to characterize the manifest distributions of observables analytically. So what did this formal exercise teach us about the three psychometric models?

The observation that accuracies and response times are dependent in the analyzed manifest distributions is a warm reminder of the fact that integrating over a common cause, or set of correlated common causes, will generate a dependency between observables that are conditionally independent. In fact, the statistical modeling of such manifest dependencies is what latent variable models are made for. Viewed in this way, it hardly seems relevant how the three latent trait models treat these dependencies locally, e.g., assuming conditional independence between observables or not, since these local properties have disappeared in the manifest distribution. Given that the manifest probabilities are all that we can ever learn from our observables, the conditional independence property may be a convenient tool to model dependencies at the latent trait level, but for the three response models it is hardly more than that.

A more sensible division of the three latent trait models appears to be the response time function that is being modeled, as it is here that we find major differences between the three response models. For example, the log-transformed response times are modeled in the HM, the residual response times or *pseudo*-response times are modeled in the SM, and in the DM the response times are modeled directly, although the latter does so in proportion to the total test time. This offers an interesting new view on response models that take response times into account, and one may wonder if there is a way to find out which function tells us the most about the unknown abilities. The manifest distributions in this paper offer one approach to address such questions.

That the conditional independence property at the latent trait level does not resonate in the practical application of the three latent trait models does not imply that this property has no impact on the manifest distribution. To see the impact of the conditional independence property at the manifest level we first note that for exponential family response models our Corollary 1 generates manifest distributions that are of the form of Eq. (13). The distribution in Eq. (13) is a prototypical example of a Markov random field (MRF; Kindermann & Snell, 1980), which is an undirected graphical model with certain conditional independence relations —known as Markov properties (e.g., Lauritzen, 2004)—that are encoded in the matrix of pairwise interactions. When accuracy and response time are independent at the latent trait level we may write this interaction matrix as

$$\begin{aligned} \varvec{\Sigma } = \begin{pmatrix} \varvec{\Sigma }_{{x}\,{x}} &{} \varvec{\Sigma }_{{x}\,{t}}\\ \varvec{\Sigma }_{{x}\,{t}}^\mathsf{T} &{}\varvec{\Sigma }_{{t}\,{t}} \end{pmatrix}, \end{aligned}$$

where $\varvec{\Sigma }_{{x} \,{x}}$ encodes the interactions between response accuracies, $\varvec{\Sigma }_{{t} \,{t}}$ the interactions between (functions of) response times, and $\varvec{\Sigma }_{{x} \,{t}}$ the interactions between the two types of observables. The division of these interactions allows us to flesh out dependencies between the two types of observables in the manifest distribution, and to specifically model any patterns of interactions that we might observe. If we wish to model such local properties we could start with models of the form of Eq. (13) or we could use higher-dimensional latent trait models (e.g., Epskamp, Kruis, & Marsman, 2015; Marsman, Maris, Bechger, & Glas, 2017; Marsman, Waldorp, & Maris, 2017).

Even though the manifest probability distributions of the DM and SM for residual times are not MRFs with respect to the two types of observables,^2^ we observed some interesting properties in their manifest distributions. In the manifest expression for the DM, for example, the associations between accuracies and response times are a function of the total time the pupil has spent on the test, an aspect that is not being modeled in any of the other manifest expressions. This property could be used to inform about the underlying strategies that pupils use, for example. The manifest expression of the SM, on the other hand, provides a new and interesting way to view an old model, the Ising model. The Ising model is an undirected graphical model that is characterized by the following distribution,

$$\begin{aligned} p(\mathbf {x}) = \frac{1}{\text {Z}} \exp \left( \mathbf {x}^\mathsf{T}\varvec{\mu } + \mathbf {x}^\mathsf{T}\varvec{\Sigma }\mathbf {x}\right) , \end{aligned}$$

where $\mathbf {x}$ is a *p*-dimensional vector of $(0,\,1)$ or $(-1,\,1)$ variables $x_i$, $\varvec{\mu }$ a *p*-dimensional vector of main effects, and $\varvec{\Sigma }$ a $p\times p$ symmetric matrix of pairwise associations between variables. Whereas the pairwise associations are fixed effects in the Ising model, the manifest distribution of the SM indicates one way to model these associations as a random effect.

One famous conjecture from Holland (1990, p. 11) is that if there are large number of items on a test, and a smooth unidimensional IRT model (for accuracies) is used, the posterior distribution of the latent trait will be approximately normal. This conjecture has inspired several publications on the posterior normality of the latent trait in the context of IRT models for response accuracy (e.g., Chang and Stout, 1993; Chang, 1996; Zhang and Stout, 1997). An interesting conclusion that Holland (1990) deduced from this conjecture, in combination with the assumption that the log-likelihoods of the *p* items can be approximated using a *p*-variate normal with a rank one covariance matrix, is that the log of the manifest distribution of accuracy is approximately of quadratic form consisting of *p* main effects and a $p \times p$ matrix of associations that was of rank one. This enticed Holland (1990) to add a second conjecture that only two parameters can be consistently estimated per item. This idea points to interesting avenues of future research, such as the asymptotic posterior normality of the latent trait in the context of IRT models for response accuracy and response times. If it is reasonable to approximate the posterior of the latent trait (or the log-likelihood function) with a normal distribution, then we can use this approximation in combination with Corollary 1 or Theorem 2 to investigate the complexity of models for response accuracy and response times, and how model complexity is impacted by the conditional independence property of the underlying latent trait model.

The latent variable distribution $g(\varvec{\zeta })$ has allowed us to express the manifest probability distributions for a large class of latent trait models, but it also generated an unexpected parameter restriction in the manifest distribution of the HM, where we found that the variance of the speed variable $v_\eta ^2$ needed to be smaller than the smallest log-normal variance $\phi _i^{-1}$. This parameter restriction follows from omitting the normalizing constants $\text {Z}_i(\varvec{\zeta })$ of the latent variable model in Eq. (7), which provides prior model structure. When a regular latent variable distribution is used—for example, a normal distribution on $\eta$—the model structure that is provided by the normalizing constants $\text {Z}_i(\varvec{\zeta })$ is integrated instead. Marsman et al. (2018) studied a similar scaling issue of the posterior distribution that results from using the latent variable distribution $g(\varvec{\zeta })$ in the context of multi-dimensional IRT (see also Marsman et al., 2017). The correspondence that we have found between our normal kernel assumption and the posterior normality assumption with the Dutch identity suggests that similar observations can be made for the prior and posterior of the latent variables in Corollary 1 of Holland (1990), Corollary 1 of Hessen (2012), and Theorem 1 in Ip (2002).

The particular restriction that is imposed on the variance of the speed variable $v_\eta ^2$ in the HM is a rather strong restriction from a substantive point of view. From the perspective of the manifest distribution, however, it might be less of an issue since $v_\eta ^2$ is simply a scaling factor for the interactions between the log-transformed response times and accuracies $\mathbf {x}$. That is, the manifest structure would not change when we absorbed $v_\eta ^2$ in the precisions and simply use the matrix of associations:

$$\begin{aligned} \varvec{\Sigma } = \begin{pmatrix} \varvec{\alpha }\varvec{\alpha }^\mathsf{T} &{} -\rho \varvec{\alpha }\, \varvec{\phi }^\mathsf{T}\\ -\rho \varvec{\phi }\,\varvec{\alpha } ^\mathsf{T}&{}\varvec{\phi }\,\varvec{\phi }^\mathsf{T} \end{pmatrix}. \end{aligned}$$

Alternatively, we may adopt a different base measure $\omega (\cdot )$ to remove the restriction.

## Appendix

### Proof of Theorem 2

The ratio of manifest distributions can be expressed as

$$\begin{aligned} \frac{p(\mathbf {x},\,\mathbf {t})}{p(\mathbf {y},\,\mathbf {w})} = \frac{\int _{\mathbb {R}} p(\mathbf {x},\,\mathbf {t} \mid \varvec{\zeta })\,f(\varvec{\zeta }) \text { d}\varvec{\zeta }}{\int _{\mathbb {R}} p(\mathbf {y},\,\mathbf {w} \mid \varvec{\zeta })\,f(\varvec{\zeta }) \text { d}\varvec{\zeta }}, \end{aligned}$$

where the latent variable model is of the form of Eq. (7), such that

$$\begin{aligned} \frac{p(\mathbf {x},\,\mathbf {t})}{p(\mathbf {y},\,\mathbf {w})} = \frac{\int _{\mathbb {R}} \prod _{i=1}^p\frac{1}{\text {Z}_i(\varvec{\zeta })} \, \text {b}_i(x_i,\,t_i) \,\exp \left( \mathbf {s}_i(x_i,\,t_i)^\mathsf{T} \varvec{\zeta }\right) \,f(\varvec{\zeta }) \text { d}\varvec{\zeta }}{\int _{\mathbb {R}} \prod _{i=1}^p\frac{1}{\text {Z}_i(\varvec{\zeta })} \, \text {b}_i(y_i,\,w_i) \,\exp \left( \mathbf {s}_i(y_i,\,w_i)^\mathsf{T} \varvec{\zeta }\right) \,f(\varvec{\zeta }) \text { d}\varvec{\zeta }}. \end{aligned}$$

Since the base measures $\text {b}_i$ do not depend on the value of the latent variables, we can place them outside of the integral ratio, and we can, moreover, nest the two integrals

$$\begin{aligned} \frac{p(\mathbf {x},\,\mathbf {t})}{p(\mathbf {y},\,\mathbf {w})} = \prod _{i=1}^p\frac{\text {b}_i(x_i,\,t_i)}{\text {b}_i(y_i,\,w_i)}\, \int _{\mathbb {R}}\frac{ \prod _{i=1}^p\frac{1}{\text {Z}_i(\varvec{\zeta })} \, \exp \left( \mathbf {s}_i(x_i,\,t_i)^\mathsf{T} \varvec{\zeta }\right) \,f(\varvec{\zeta })}{\int _{\mathbb {R}} \prod _{i=1}^p \frac{1}{\text {Z}_i(\varvec{\zeta })} \,\exp \left( \mathbf {s}_i(y_i,\,w_i)^\mathsf{T} \varvec{\zeta }\right) \,f(\varvec{\zeta }) \text { d}\varvec{\zeta }}\text { d}\varvec{\zeta }. \end{aligned}$$

We next multiply the integrand of the outer integral with the factor

$$\begin{aligned} 1 = \prod _{i=1}^p\frac{\exp \left( \mathbf {s}_i(y_i,\,w_i)^\mathsf{T} \varvec{\zeta }\right) }{\exp \left( \mathbf {s}_i(y_i,\,w_i)^\mathsf{T} \varvec{\zeta }\right) }, \end{aligned}$$

as follows

$$\begin{aligned} \frac{p(\mathbf {x},\,\mathbf {t})}{p(\mathbf {y},\,\mathbf {w})}&= \prod _{i=1}^p \frac{\text {b}_i(x_i,\,t_i)}{\text {b}_i(y_i,\,w_i)} \int _{\mathbb {R}}\prod _{i=1}^p\frac{\exp \left( \mathbf {s}_i(y_i,\,w_i)^\mathsf{T} \varvec{\zeta }\right) }{\exp \left( \mathbf {s}_i(y_i,\,w_i)^\mathsf{T} \varvec{\zeta }\right) } \\&\quad \frac{\prod _{i=1}^p\frac{1}{\text {Z}_i(\varvec{\zeta })} \exp \left( \mathbf {s}_i(x_i,\,t_i)^\mathsf{T} \varvec{\zeta }\right) f(\varvec{\zeta })}{\int _{\mathbb {R}} \prod _{i=1}^p\frac{1}{\text {Z}_i(\varvec{\zeta })} \exp \left( \mathbf {s}_i(y_i,\,w_i)^\mathsf{T} \varvec{\zeta } \right) f(\varvec{\zeta }) \text { d}\varvec{\zeta }}\text { d}\varvec{\zeta } \\&= \prod _{i=1}^p \frac{\text {b}_i(x_i,\,t_i)}{\text {b}_i(y_i,\,w_i)} \int _{\mathbb {R}}\prod _{i=1}^p\frac{ \exp \left( \mathbf {s}_i(x_i,\,t_i)^\mathsf{T} \varvec{\zeta }\right) }{ \exp \left( \mathbf {s}_i(y_i,\,w_i)^\mathsf{T} \varvec{\zeta }\right) } \\&\quad \frac{\prod _{i=1}^p\frac{1}{\text {Z}_i(\varvec{\zeta })} \exp \left( \mathbf {s}_i(y_i,\,w_i)^\mathsf{T} \varvec{\zeta } \right) f(\varvec{\zeta }) }{\int _{\mathbb {R}} \prod _{i=1}^p\frac{1}{\text {Z}_i(\varvec{\zeta })} \exp \left( \mathbf {s}_i(y_i,\,w_i)^\mathsf{T} \varvec{\zeta } \right) f(\varvec{\zeta }) \text { d}\varvec{\zeta }}\text { d}\varvec{\zeta }, \end{aligned}$$

so that we can now recognize the second factor in the integrand as the posterior distribution

$$\begin{aligned} f\left( \varvec{\zeta } \,\bigg |\, \mathbf {S} = \sum _i\mathbf {s}_i(y_i,\,w_i)\right) = \frac{\prod _{i=1}^p\frac{1}{\text {Z}_i(\varvec{\zeta })} \exp \left( \mathbf {s}_i(y_i,\,w_i)^\mathsf{T} \varvec{\zeta } \right) f(\varvec{\zeta }) }{\int _{\mathbb {R}} \prod _{i=1}^p\frac{1}{\text {Z}_i(\varvec{\zeta })} \exp \left( \mathbf {s}_i(y_i,\,w_i)^\mathsf{T} \varvec{\zeta } \right) f(\varvec{\zeta }) \text { d}\varvec{\zeta }}, \end{aligned}$$

and we can express the ratio in the following form

$$\begin{aligned} \frac{p(\mathbf {x},\,\mathbf {t})}{p(\mathbf {y},\,\mathbf {w})}&= \prod _{i=1}^p \frac{\text {b}_i(x_i,\,t_i)}{\text {b}_i(y_i,\,w_i)} \int _{\mathbb {R}}\prod _{i=1}^p\frac{ \exp \left( \mathbf {s}_i(x_i,\,t_i)^\mathsf{T} \varvec{\zeta }\right) }{ \exp \left( \mathbf {s}_i(y_i,\,w_i)^\mathsf{T} \varvec{\zeta }\right) }\, f\left( \varvec{\zeta } \,\bigg |\, \mathbf {S} = \sum _i\mathbf {s}_i(y_i,\,w_i)\right) \text { d}\varvec{\zeta } \\&= \prod _{i=1}^p \frac{\text {b}_i(x_i,\,t_i)}{\text {b}_i(y_i,\,w_i)} \int _{\mathbb {R}} \prod _{i=1}^p \exp \left( \left[ \mathbf {s}_i(x_i,\,t_i) - \mathbf {s}_i(y_i,\,w_i)\right] ^\mathsf{T} \varvec{\zeta }\right) \\&\quad f\left( \varvec{\zeta } \,\bigg |\, \mathbf {S} = \sum _i\mathbf {s}_i(y_i,\,w_i)\right) \text { d}\varvec{\zeta } \\&{= \prod _{i=1}^p \frac{\text {b}_i(x_i,\,t_i)}{\text {b}_i(y_i,\,w_i)} \mathbb {E}\left( \left. \prod _{i=1}^p\exp \left( \left[ \mathbf {s}_i(x_i,\,t_i) - \mathbf {s}_i(y_i,\,w_i)\right] ^\mathsf{T} \varvec{\zeta }\right) \,\right| \,\mathbf {S} = \sum _i\mathbf {s}_i(y_i,\,w_i)\right) ,} \end{aligned}$$

which completes our proof.

## Footnotes

1 Alternatively, one may choose an item *i* and constrain $\beta _i$ and $\xi _i$ to zero and constrain $\alpha _i$ to one for this item.

2 Observe, however, that for both models the conditional distribution $p(\mathbf {x} \mid \mathbf {t})$ is an MRF, because they share the Markov property that when the association between the accuracy of an item *i* and an item *j* is equal to zero, these two variables are independent given the accuracy on the remaining items. Incidentally, for the manifest expressions of both the SM and the DM the associated conditional distribution is another instance of the Ising model.

## References

[CR1] Anderson C, Vermunt J (2000). Log-multiplicative association models as latent variable models for nominal and/or ordinal data. Sociological Methodology.

[CR2] Anderson C, Yu H-T (2007). Log-multiplicative association models as item response models. Psychometrika.

[CR3] Bolsinova M, De Boeck P, Tijmstra J (2017). Modelling conditional dependence between response time and accuracy. Psychometrika.

[CR4] Bolsinova M, Maris G (2016). A test for conditional independence between response time and accuracy. British Journal of Mathematical and Statistical Psychology.

[CR5] Bolsinova M, Tijmstra J (2016). Posterior predictive checks for conditional independence between response time and accuracy. Journal of Educational and Behavioural Statistics.

[CR6] Bolsinova M, Tijmstra J, Molenaar D (2017). Response moderation models for conditional dependence between response time and response accuracy. British Journal of Mathematical and Statistical Psychology.

[CR7] Chang H (1996). The asymptotic posterior normality of the latent trait for polytomous IRT models. Psychometrika.

[CR8] Chang H, Stout W (1993). The asymptotic posterior normality of the latent trait in an IRT model. Psychometrika.

[CR9] Chen Y, Li X, Liu J, Ying Z (2018). Robust measurement via a fused latent and graphical item response theory model. Psychometrika.

[CR10] Cox D, Miller H (1970). The theory of stochastic processes.

[CR11] Cressie N, Holland P (1983). Characterizing the manifest probabilities of latent trait models. Psychometrika.

[CR12] Efron B (1975). Defining the curvature of a statistical problem (with applications to second order efficiency). The Annals of Statistics.

[CR13] Efron B (1978). The geometry of exponential families. The Annals of Statistics.

[CR14] Epskamp S, Kruis J, Marsman M (2017). Estimating psychopathological networks: Be careful what you wish for. PLoS ONE.

[CR15] Epskamp S, Maris G, Waldorp L, Borsboom D, Irwing P, Hughes D, Booth T (2018). Network psychometrics. Handbook of psychometrics.

[CR16] Fox J-P, Klein Entink R, van der Linden W (2007). Modeling of responses and response times with the package CIRT. Journal of Statistical Software.

[CR17] Hessen D (2012). Fitting and testing conditional multinormal partial credit models. Psychometrika.

[CR18] Holland P (1990). The Dutch identity: A new tool for the study of item response models. Psychometrika.

[CR19] Ip E (2002). Locally dependent latent trait model and the Dutch identity revisited. Psychometrika.

[CR20] Ising E (1925). Beitrag zur theorie des ferromagnetismus. Zeitschrift für Physik.

[CR21] Kac, M. (1968). Mathematical mechanisms of phase transitions. InM. Chrétien, E. Gross, & S. Deser (Eds.), Statistical physics: Phase transitions and superfluidity, Vol. 1, Brandeis University Summer Institute in Theoretical Physics (pp. 241–305). New York: Gordon and Breach Science Publishers.

[CR22] Kindermann R, Snell JL (1980). Markov random fields and their applications.

[CR23] Klein Entink R, van der Linden W, Fox J-P (2009). A Box–Cox normal model for response times. British Journal of Mathematical and Statistical Psychology.

[CR24] Lauritzen S (2004). Graphical models.

[CR25] Lenz W (1920). Beiträge zum verständnis der magnetischen eigenschaften in festen körpern. Physikalische Zeitschrift.

[CR26] Maris G, van der Maas H (2012). Speed-accuracy response models: Scoring rules based on response time and accuracy. Psychometrika.

[CR27] Marsman M, Borsboom D, Kruis J, Epskamp S, van Bork R, Waldorp L, Maris G (2018). An introduction to network psychometrics: Relating Ising network models to item response theory models. Multivariate Behavioral Research.

[CR28] Marsman M, Maris G, Bechger T, Glas C (2015). Bayesian inference for low-rank Ising networks. Scientific Reports.

[CR29] Marsman, M., Tanis, C., Bechger, T., & Waldorp, L. (2019). Network psychometrics in educational practice. Maximum likelihood estimation of the Curie-Weiss model. Manuscript submitted for publication.

[CR30] Marsman M, Waldorp L, Maris G (2017). A note on large-scale logistic prediction: Using an approximate graphical model to deal with collinearity and missing data. Behaviormetrika.

[CR31] McCullagh P (1994). Exponential mixtures and quadratic exponential families. Biometrika.

[CR32] Molenaar D, Tuerlinckx F, van der Maas H (2015). A bivariate generalized linear item response theory modeling framework to the analysis of responses and response times. Multivariate Behavioural Research.

[CR33] Molenaar D, Tuerlinckx F, van der Maas H (2015). A generalized linear factor model approach to the hierarchical framework for responses and response times. British Journal of Mathematical and Statistical Psychology.

[CR34] Ratcliff R (1978). A theory of memory retrieval. Psychological Review.

[CR35] Tjur T (1982). A connection between Rasch’s item analysis model and a multiplicative poisson model. Scandinavian Journal of Statistics.

[CR36] Tuerlinckx F, De Boeck P (2005). Two interpretations of the discrimination parameter. Psychometrika.

[CR37] van der Linden W (2006). A lognormal model for response times on test items. Journal of Educational and Behavioural Statistics.

[CR38] van der Linden W (2007). A hierarchical framework for modeling speed and accuracy on test items. Psychometrika.

[CR39] van der Linden W, Glas C (2010). Statistical tests of conditional independence between responses and/or response times on test items. Psychometrika.

[CR40] van Rijn P, Ali U (2017). A comparison of item response models for accuracy and speed of item responses with applications to adaptive testing. British Journal of Mathematical and Statistical Psychology.

[CR41] van Rijn P, Ali U (2017). A generalized speed-accuracy response model for dichotomous items. Psychometrika.

[CR42] Zhan P, Jiao H, Liao D (2018). Cognitive diagnosis modelling incorporating item response times. British Journal of Mathematical and Statistical Psychology.

[CR43] Zhang J, Stout W (1997). On Holland’s Dutch identity conjecture. Psychometrika.

